# The influence of tDCS on perceived bouncing/streaming

**DOI:** 10.1007/s00221-022-06505-5

**Published:** 2022-11-10

**Authors:** Maximilian A. Friehs, Marlena J. Stegemann, Simon Merz, Christoph Geißler, Hauke S. Meyerhoff, Christian Frings

**Affiliations:** 1 Leibniz-Institut für Wissensmedien, Tübingen, Germany; 2 Lise-Meitner Research Group Cognition and Plasticity, Max-Planck-Institute for Human Cognitive and Brain Science, Leipzig, Germany; 3 University of Trier, Trier, Germany; 4 University of Erfurt, Erfurt, Germany; 5 School of Psychology, University College Dublin, Dublin, Ireland

**Keywords:** Crossmodal integration, Multisensory perception, tDCS, Bouncing/streaming

## Abstract

Processing ambiguous situations is a constant challenge in everyday life and sensory input from different modalities needs to be integrated to form a coherent mental representation on the environment. The bouncing/streaming illusion can be studied to provide insights into the ambiguous perception and processing of multi-modal environments. In short, the likelihood of reporting bouncing rather than streaming impressions increases when a sound coincides with the moment of overlap between two moving disks. Neuroimaging studies revealed that the right posterior parietal cortex is crucial in cross-modal integration and is active during the bouncing/streaming illusion. Consequently, in the present study, we used transcranial direct current stimulation to stimulate this brain area. In the active stimulation conditions, a 9 cm^2^ electrode was positioned over the P4-EEG position and the 35 cm^2^ reference positioned over the left upper arm. The stimulation lasted 15 min. Each participant did the bouncing/streaming task three times: before, during and after anodal or sham stimulation. In a sample of *N* = 60 healthy, young adults, we found no influence of anodal tDCS. Bayesian analysis showed strong evidence against tDCS effects. There are two possible explanations for the finding that anodal tDCS over perceptual areas did not modulate multimodal integration. First, upregulation of multimodal integration is not possible using tDCS over the PPC as the integration process already functions at maximum capacity. Second, prefrontal decision-making areas may have overruled any modulated input from the PPC as it may not have matched their decision-making criterion and compensated for the modulation.

## Introduction

The perception of our environment is formed by sensory input from different modalities. This input has to be integrated into a coherent perception. This is especially challenging in ambiguous situations. A good example of such an ambiguity is the bouncing/streaming display that is based on the paradigm developed by Metzger (1934). In this display, two identical disks move horizontally toward each other, overlap in the center of the screen, and then move apart again. The ambiguity of this display emerges by two different possible interpretations that are equally representative of two different perceptions in the real world. One interpretation is the impression of two disks bouncing off each other while the other interpretation is two disks streaming past each other. Typically, observers perceive this display as two disks streaming past each other but when a brief tone coincides with the moment of overlap, the perception changes to bouncing impressions (Sekuler et al. 1997). This display, therefore, perfectly enables the investigation of the integration of sensory input from different modalities.

Previous studies showed an involvement of multimodal brain areas in the bouncing-streaming illusion (Ashbridge 1997; Bushara et al. 2003; Heinen et al. 2011; Sereno and Huang 2014). In the present study, we investigated the effect of anodal tDCS on multimodal brain areas. We hypothesized that anodal tDCS on the right posterior parietal area increases the multisensory integration of the visual and auditory stimulus and therefore leads to an increase in the bouncing-streaming illusion.

## The bouncing-streaming illusion in the brain

Neuroimaging studies provided evidence that, generally speaking, the right posterior parietal cortex (PPC) is involved in cross-modal binding and specifically during the bouncing-streaming illusion (Ashbridge 1997; Bushara et al. 2003; Heinen et al. 2011; Sereno and Huang 2014). Thus, the right PPC plays a crucial role in the integration of multimodal stimuli. Furthermore, a study using magnetoencephalography reported that early activation of the attention-related brain areas and subsequent involvement of the multisensory areas is associated with an increase with a bouncing-percept in the bouncing-streaming illusion, which further supports the role of the right PPC in multisensory integration (Zvyagintsev et al. 2011).

One way to investigate the neurophysiological underpinnings of the bouncing-streaming illusion is the use of non-invasive brain stimulation (NIBS) techniques, such as transcranial magnetic stimulation (TMS) and transcranial direct current stimulation (tDCS). One of the most important differences between TMS and tDCS is the ability to directly induce action potentials. While TMS utilizes a rapidly changing magnetic field to directly induce action potentials in the targeted brain area, tDCS induces sub-threshold modulation of the membrane potentials during the stimulation (Bergmann and Hartwigsen 2020). Further, tDCS may induce changes in neurotransmitter activity after the stimulation has ceased.

A recent TMS study targeted the right PPC using offline stimulation (i.e., stimulation before the task performance) to modulate the bouncing judgment (Maniglia et al. 2012). Their results showed that the amount of bouncing responses decreases after disruption of the right PPC using TMS. These results are in line with the current models of this illusion; specifically by disrupting the right PPC, the integration of the audio–visual is perturbed and consequently the bouncing judgment reduced. With that being said, it remains unclear whether or not it is also possible to modify the bouncing-streaming illusion by upregulating the right PPC in a way that may enhance perceptual effect. One way to increase activity in a brain area and potentially subsequently increase the percept of the illusion is anodal tDCS. Previous research has shown that tDCS effects are time and polarity specific (Friehs and Frings 2019; Jamil et al. 2019; Stagg et al. 2011a, b). Crucial for the present study are the differential effects of online and offline anodal stimulation. Online anodal tDCS can induce subthreshold changes in the resting membrane potential, which in turn can alter the spontaneous firing rate of neurons and modulate their response to incoming signals. In detail, during anodal tDCS, a Na + influx into the cell leads to an initial depolarization, which in turn opens voltage-gated Ca^2+^ channels of the N-methyl-D-aspartate receptor. These tonic shifts in membrane potentials will influence neuronal excitability in a stimulation polarity-dependent way. In contrast, offline anodal tDCS effects are most likely driven by an activation of the NMDA receptors caused by a decrease in GABAergic tone. More specifically, a decrease in GABA concentrations within the stimulated area under the electrode has been observed after anodal tDCS. The lasting after-effects of tDCS may reflect LTP-like plasticity (Jamil et al. 2017; Stagg et al. 2011a, b; Stagg et al. 2009).

## The present study and hypothesis

In the present study, we contrasted the effects of anodal and sham tDCS over the right PPC. We employed a repeated measures design in which two groups (anodal or sham tDCS) completed the bouncing-streaming illusion task three times during their respective stimulation session: one before, during and after either anodal or sham stimulation. We hypothesize that an upregulation via anodal tDCS of the right PPC will lead to an increase in the bounce illusion, due to an increase in multisensory integration of the visual and auditory stimulus.

To reiterate, in contrast to Maniglia and colleagues (2012), this study aims to increase multimodal integration via tDCS instead of perturbing this process using TMS. Consequently, if tDCS has an influence on the bouncing-streaming judgment, the automatic multimodal integration process may not operate at maximum capacity in laboratory settings.

## Methods

We have preregistered the experiment at the Open Science Framework (https://osf.io/ukdc8).

### Participants

Sixty students from the University of Trier (18–31 years, 47 females, 10 males, 1 other, 2 NA) completed the experiment in exchange for payment or partial course credit. Thirty students were each assigned to either active, anodal or sham tDCS. All participants reported to be right-handed.

### Apparatus, stimuli, and procedure

Participants were seated in front of a 19-in. color monitor with a viewing distance of 65 cm in a normally lit room. Participants responded only using their right hand by pressing one of two marked keys on a keyboard in front of the monitor. The stimuli were programmed using PsychoPy3 (Peirce et al. 2019).

**Fig. 1:**
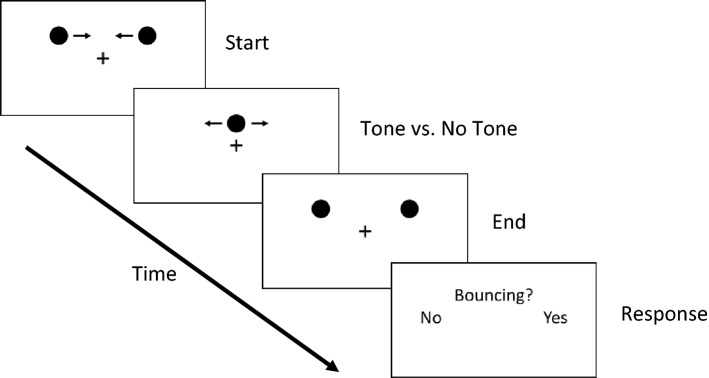
An illustration of the bouncing/streaming task. Two disks moved toward each other, fully overlapped, and then moved apart again. In half of the trials, a tone coincided with the moment of overlap of the disks. Afterward, participants indicated whether or not they perceived the disks as bouncing off each other

At the beginning of each trial, a central fixation cross was presented, and the participants were instructed to fixate it for the entire trial. After 300 ms, two white disks (0.5 degree in diameter) appeared 5 degree above the center in the left and right visual fields against a black background. Immediately after onset, the disks started moving toward each other along a horizontal path at a constant speed of 10 degree/s until they fully overlapped above the fixation cross after 1000 ms of motion. Following this, the disks moved apart again for another interval of 1000 ms. In half of the trials, a 440 Hz pure sine wave tone was presented for 100 ms at the moment of overlap of the disks. At the end of each trial, the participants indicated whether or not they perceived the disks as bouncing off each other by pressing the respective key on a keyboard (see Fig. 1) Each participant completed six practice trials followed by three experimental sessions. Each session consisted of 108 experimental trials (54 per condition). Tone and no-tone trials were randomized. In three experimental sessions, each participant completed the bouncing/streaming illusion task before, during (“online”) and after (“offline”) either active or sham stimulation. The sessions tasks were completed in one sitting on the same day with either active or sham stimulation, which was varied between-subjects. In the anodal stimulation condition, current was applied. In the sham stimulation condition, the current was only applied during a short ramp-up/-down phase at the start and end of the stimulation period. In general, the ramp-up/down phase helps participants to experience a smooth transition, instead of a harsher sensation when the stimulation is switched on or off. Additionally, this procedure evokes a physical sensation for participants but does not provide enough stimulation as to actually modulate the underlying brain area. In all other aspects of the experimental procedure, the sham condition was identical to the anodal condition (e.g., in both conditions, the electrodes were attached).

#### Transcranial direct current stimulation tDCS

**Fig. 2:**
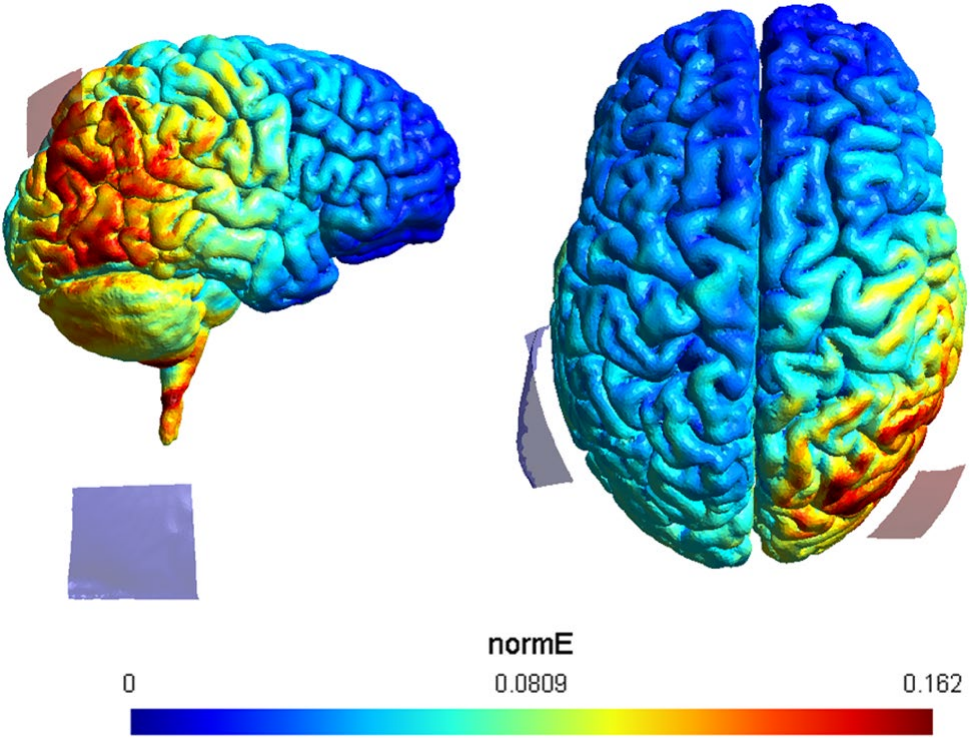
Transcranial DC stimulation (tDCS) electrodes were positioned over P4 and left deltoid. Note that the body itself is not displayed, but the relative position of the blue electrode (i.e., the cathode) represents the left deltoid. To the left, a view of the right hemisphere is presented, and to the right, a top view of the brain. Current flow simulation done with SimNIBS

Direct current was provided by a 4-channel-DC-stimulator (NeuroConn, Ilmenau). In both anodal and sham stimulation condition, the participants were fitted with two electrodes. The 3 × 3 cm anode was positioned over the right PPC (P4; 10–20 EEG position), while the 7 × 5 cm cathode was mounted over the left upper arm. A constant current of 0.7 mA was applied for a total of 15 min. Previous studies showed that after-effects of tDCS may occur after only 7 min of stimulation (e.g., Nitsche et al. 2005). This resulted in a current density of 0.078 mA/cm^2^. The stimulation included a ramp-up/-down phase of 30 s each at the start and end of the stimulation phase. The sham condition only included the aforementioned ramp-up/-down phases at the start and end of the stimulation time. Upon entering the laboratory, participants were randomly assigned to a stimulation condition. The stimulation was controlled via a panel PC, and current flow patterns over the stimulated brain regions were validated using the software HD-Explore (Soterix Medical Inc., New York) and SimNIBS (Saturnino et al. 2019). For a visual representation of the current flow simulation with SimNIBS, see Fig. 2.

## Results

**Fig. 3:**
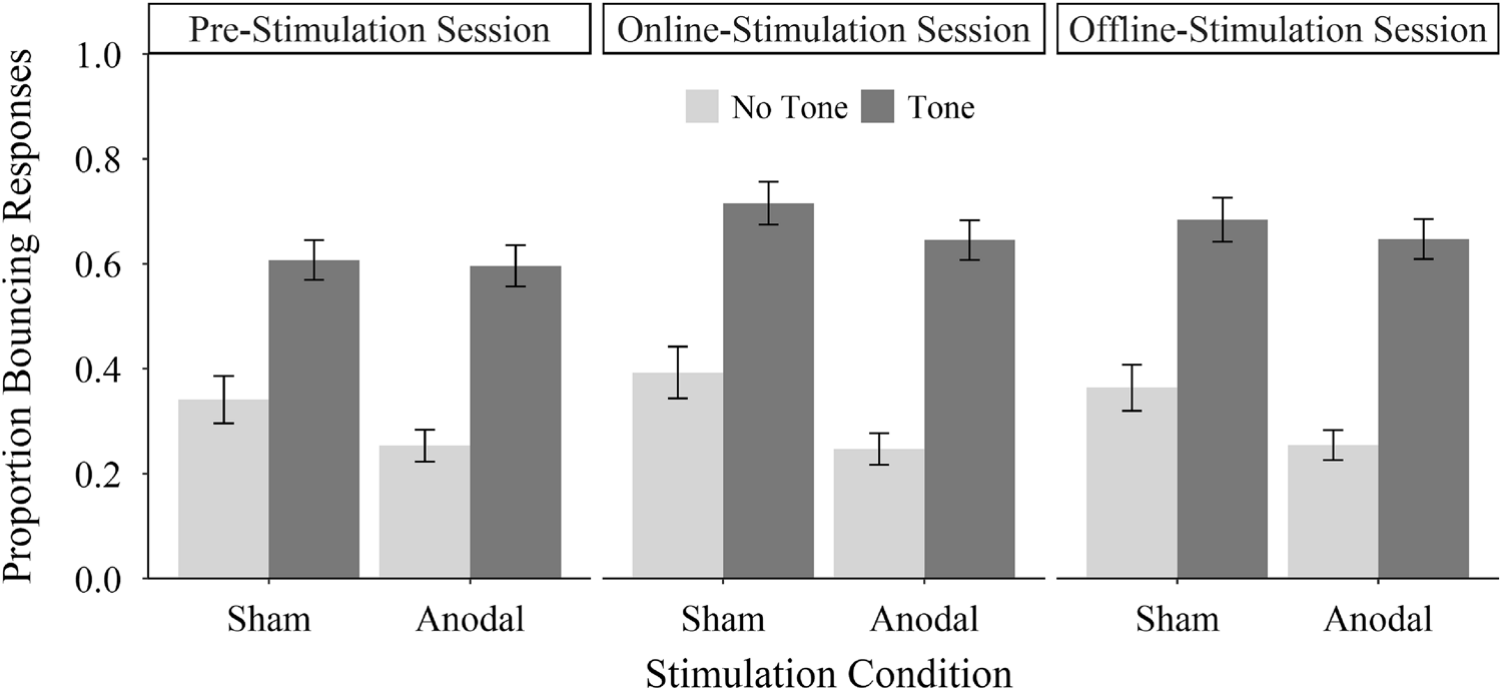
Mean proportion of bouncing responses as a function of stimulation condition (sham vs. anodal), session (pre-stimulation vs. online stimulation vs. offline stimulation) and trial type (tone present vs. absent). Error bars represent ± 1 standard error of the mean

**Fig. 4:**
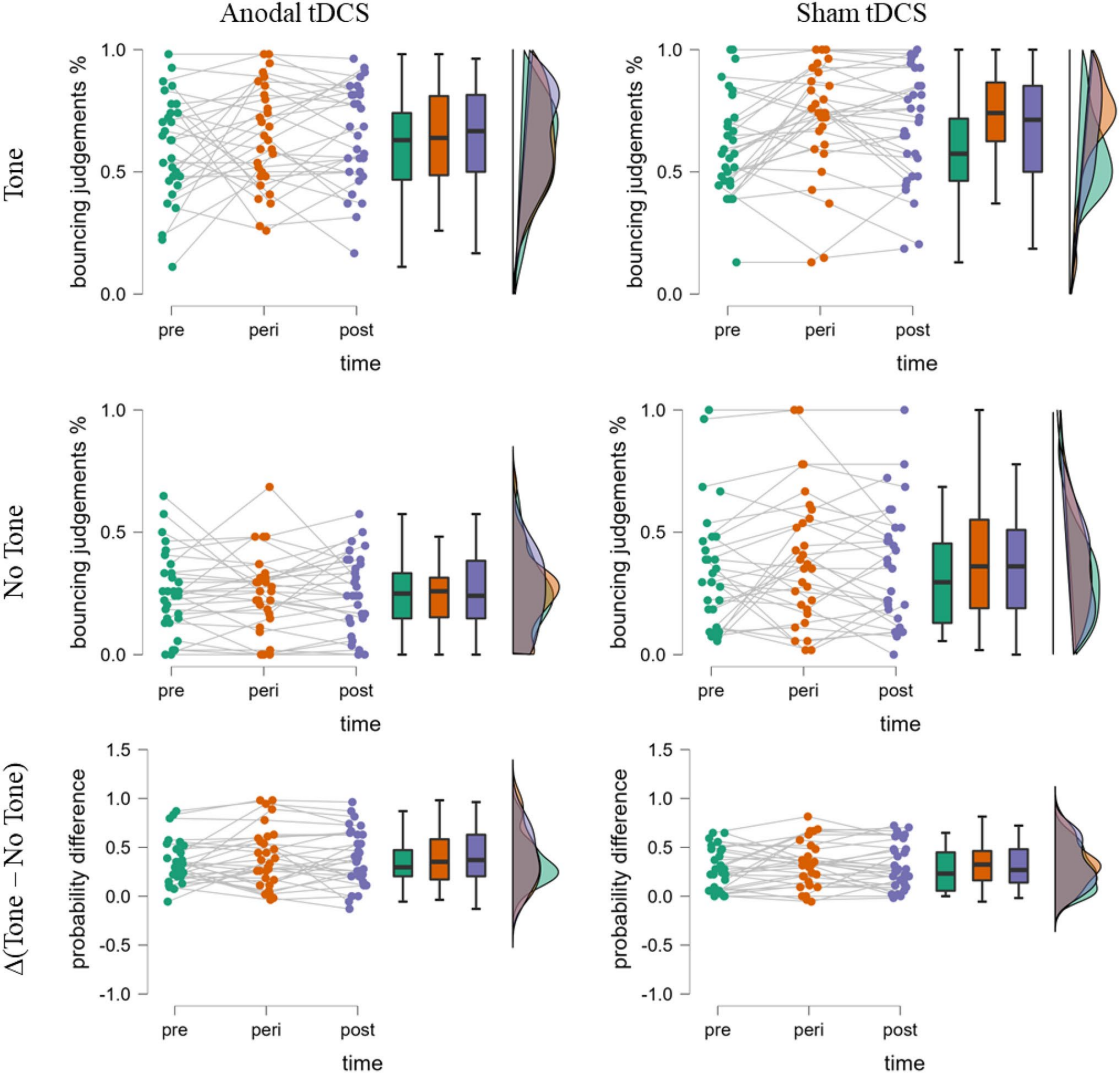
Bouncing/streaming judgment distribution depending on stimulation condition (anodal vs. sham) and trial type (tone vs no-tone). Each study participant is separately represented by a dots connected by a gray line. Visuals created with JASP

We calculated for each participant the proportion of bouncing responses for each experimental condition. To investigate whether anodal tDCS increased the bouncing/streaming illusion, we conducted a 2 (stimulation condition, between-subjects: sham vs. anodal) × 3 (session, within-subject: pre-stimulation vs. online stimulation vs. offline stimulation) × 2 (trial type, within-subject: tone present vs. absent) mixed analysis of variance (ANOVA) with the proportion of bouncing responses as dependent variable. The results showed a higher proportion of bouncing responses with than without coinciding tones, replicating the bouncing/streaming illusion, *F*(1,58) = 147.49, *p* < 0.001, ${\eta }_{\mathrm{P}}^{2}$ = 0.72. Moreover, the results showed an effect of the session, *F*(2,116) = 3.36, *p* = 0.049, ${\eta }_{\mathrm{P}}^{2}$ = 0.05, with a lower proportion of bouncing responses in the pre-stimulation session than in the online stimulation session, *t*(59) = 2.29, *p* = 0.026, *d*_*z*_ = 0.30. Furthermore, there was an effect of the stimulation condition with a higher proportion of bouncing responses in the sham stimulation condition than in the anodal stimulation condition, *F*(1,58) = 4.02, *p* = 0.050, ${\eta }_{\mathrm{P}}^{2}$ = 0.06. Importantly, however, we did not find the expected three-way interaction, *F*(2,116) < 0.01, *p* = 0.996, ${\eta }_{\mathrm{P}}^{2}$ < 0.01 (see Fig. 3). Therefore, there was no modulation of anodal tDCS on the bouncing/streaming illusion. No further main effects or interactions reached significance, *p*s > 0.05. Additionally, the effects did not differ on an individual level (see Fig. 4). To assess the overlap between conditions, the difference between tone and no-tone trials depending on the tDCS condition and time-point was analyzed with the Overlapping-Package in R (Pastore, 2018). The overlap between stimulation-based distributions was 70% before the stimulation, 78% during the stimulation and 76% after the stimulation.

*Bayesian Analysis*. To establish further support for the null result, we employed Bayesian analysis and repeated the frequentist analysis (van den Bergh et al. 2020; Wagenmakers et al. 2018a, b; Wagenmakers et al. 2018a, b). We utilized matched models suggested by Sebastiaan Mathôd to simplify interpretation of the effects (van den Bergh et al. 2020), which contrasts all models with a certain effect to all models without that effect. For example, to solve for the interaction of variables A x B in a two-factorial design, the model with this interaction is compared to models with the main effects of A and B. The results show that for the present study, there is strong evidence against the tDCS effects and for the null hypothesis as indicated by the BF_exclusion_ = 11.05 for the three-way interaction of auditory cue x tDCS condition x session.

## Discussion

In the present study, we investigated the influence of anodal and sham tDCS over the right PPC on perceived bounding/streaming. Overall, a strong increase in the proportion of bouncing responses was observed when a tone was presented simultaneous with the overlapping of the two disks. This convincingly replicated previous reports of the bouncing/streaming illusion (e.g., Meyerhoff and Suzuki 2018; Sekuler et al. 1997). Crucially, there was no influence of anodal tDCS on the bouncing-streaming illusion. In line with the frequentist analysis, the Bayesian analysis also showed strong evidence against a modulatory influence of the anodal tDCS stimulation. Thus, neither online stimulation nor off-line stimulation exerted any influence on the perceived bouncing-streaming illusion. One possible explanation of our results is that the targeted brain area is not directly involved in generating the bouncing/streaming illusion or only plays a minor role in the network responsible for the illusion. This may have implications for theories addressing the origin of the bouncing-streaming illusion. Some recent findings suggest that the bouncing impression arise at a perceptual level (Meyerhoff and Scholl 2018; Meyerhoff and Suzuki 2018; for different views see Grassi and Casco 2009, 2010; Grove et al. 2012; Zeljko and Grove 2021). Another possibility is that the area already had reached its processing peak and thus increasing neural processing via anodal tDCS could not have an up-regulatory effect. Future studies may investigate this further utilizing different paradigms with multiple stimuli that may be bound together and not just one pair as in the current study. Two opposing results may be expected if brain stimulation can actually up-regulate a perceptual area and modify the integration process. Either, the temporal binding window may decrease and only few stimuli are bound together. Or, the temporal increases and more stimuli (compared to baseline) are integrated.

### Limitations and alternative explanations

Although the results may be considered as evidence against the possibility of modulating the bouncing-streaming effect via anodal stimulation of the right PPC, there are also several limitations that need to be considered. First, since no additional neuroimaging was employed, we cannot conclusively prove that tDCS impacted brain activity in all participants, given that inter-individual differences may modulate tDCS effects and that the same stimulation protocol may yield different results in different samples (Chew et al. 2015; Friehs, Frings, et al. 2021; Horvath et al. 2014; Krause and Cohen Kadosh 2014). Second, ideally the study would have employed a complete within-subjects design and additionally investigated the effect of cathodal tDCS (for example Friehs et al. 2021a, b, 2022 for a discussion of the topic). This would have had the additional benefit of being more comparable to Maniglia and colleagues (2012), who used TMS to perturb the right PPC. However, a combined anodal and cathodal tDCS design would also have its drawbacks apart from a reduced practicability. For example, in between the individual testing sessions participants could have relatively simply identified the premise of the task and looked into the illusion itself, which would have had detrimental effects on subsequent task performance (see Meyerhoff and Scholl 2018, for an alternative measure). Third, our results are somewhat in contrast to the results reported by Maniglia and colleagues (2012). They report that disruption of the right PPC via 1 Hz offline TMS, reduced the bouncing/streaming illusion. In conjunction with our results, it may be conjectured that TMS impacted the bouncing/streaming percept by modulation of downstream network nodes and not only via modification of right PPC activity. Specifically, prefrontal areas are also involved in the illusion (Bushara et al. 2003; Zhao et al. 2018). For example, Zhao and colleagues (2018) provide evidence of activity in a frontal network after the two disks met and a sound cue was played and similarly Bushara and colleagues (2003) show that the left dorsolateral prefrontal cortex has increased activity in ‘bouncing’ trials compared to ‘streaming’ trials. This may reflect an involvement of prefrontal areas in a decision-making process, after the stimulus display is processed. In the present study as well as in Maniglia et al. (2012), the stimulation of the right PPC targets an area in the perceptual stage of processing, and the processing outcome in the PPC will serve as incoming signals to the frontal areas. Thus, it may be that frontal areas tried to compensate for modulated signals from the PPC that did not match their decision-making criterion. In this way, the prefrontal areas may compensate for the changed processing in the right PPC due to tDCS (for a discussion on compensatory processes see Friehs et al. 2021a, b; Friehs et al. 2020; Hartwigsen 2018).

Another possible limitation of the study stems from the fact that a within-study design was employed. Although, in general within-study design is preferable as inter-individual variability is reduced, the participants in the present study however could have become set in their response pattern in the pre-tDCS session and strived for consistency in the later stages of the experiment. This could have counteracted the potential stimulation effect, but on the flipside, if a tDCS effect was observed in the present study, we could have been sure that the effect was not due to the inter-individual variability.

## Conclusion

In conclusion, in a study investigating both online and offline tDCS on the perception of the bouncing/streaming illusion, we did not observe any effects of stimulation on bouncing/streaming judgements. In fact, the data revealed evidence in favor of the null hypothesis. Although there are a number of potential explanations for this result, the most theoretically intriguing is that the present results reflect a robustness of the perceptual multimodal integration system that already is functioning as efficiently as possible.

## Data Availability

The data are available under https://osf.io/ukdc8.
